# IFN-α confers epigenetic regulation of HBV cccDNA minichromosome by modulating GCN5-mediated succinylation of histone H3K79 to clear HBV cccDNA

**DOI:** 10.1186/s13148-020-00928-z

**Published:** 2020-09-07

**Authors:** Ying Yuan, Hongfeng Yuan, Guang Yang, Haolin Yun, Man Zhao, Zixian Liu, Lina Zhao, Yu Geng, Lei Liu, Jiapei Wang, Huihui Zhang, Yufei Wang, Xiao-dong Zhang

**Affiliations:** Nankai University, 94 Weijin Road, Tianjin, 300071 People’s Republic of China

**Keywords:** Interferon-α, HBV, HBV cccDNA minichromosome, Histone succinylation, GCN5, Epigenetic regulation

## Abstract

**Background:**

Hepatitis B virus covalently closed circular DNA (HBV cccDNA) is assembled by histones and non-histones into a chromatin-like cccDNA minichromosome in the nucleus. The cellular histone acetyltransferase GCN5, displaying succinyltransferase activity, is recruited onto cccDNA to modulate HBV transcription in cells. Clinically, IFN-α is able to repress cccDNA. However, the underlying mechanism of IFN-α in the depression of cccDNA mediated by GCN5 is poorly understood. Here, we explored the effect of IFN-α on GCN5-mediated succinylation in the epigenetic regulation of HBV cccDNA minichromosome.

**Results:**

Succinylation modification of the cccDNA minichromosome has been observed in HBV-infected human liver-chimeric mice and HBV-expressing cell lines. Moreover, histone H3K79 succinylation by GCN5 was identified in the system. Interestingly, the mutant of histone H3K79 efficiently blocked the replication of HBV, and interference with GCN5 resulted in decreased levels of HBV DNA, HBsAg, and HBeAg in the supernatant from de novo HBV-infected HepaRG cells. Consistently, the levels of histone H3K79 succinylation were significantly elevated in the livers of HBV-infected human liver-chimeric mice. The knockdown or overexpression of GCN5 or the mutant of GCN5 could affect the binding of GCN5 to cccDNA or H3K79 succinylation, leading to a change in cccDNA transcription activity. In addition, Southern blot analysis validated that siGCN5 decreased the levels of cccDNA in the cells, suggesting that GCN5-mediated succinylation of histone H3K79 contributes to the epigenetic regulation of cccDNA minichromosome. Strikingly, IFN-α effectively depressed histone H3K79 succinylation in HBV cccDNA minichromosome in de novo HepG2-NTCP and HBV-infected HepaRG cells.

**Conclusions:**

IFN-α epigenetically regulates the HBV cccDNA minichromosome by modulating GCN5-mediated succinylation of histone H3K79 to clear HBV cccDNA. Our findings provide new insights into the mechanism by which IFN-α modulate the epigenetic regulation of HBV cccDNA minichromosome.

## Introduction

Chronic hepatitis B virus (HBV) infection is a leading cause of hepatitis, cirrhosis, and liver cancer, resulting in an estimated 650,000 deaths per year [1–3]. HBV, as a small enveloped DNA virus, replicates its genome in the cytoplasm via reverse transcription of the encapsidated pregenomic RNA (pgRNA) into a viral relaxed circular DNA (rcDNA) [4–6]. Upon infection, the encapsidated rcDNA is imported into the nucleus, where it is converted into covalently closed circular DNA (cccDNA), which serves as the template for viral transcription and secures HBV persistence [7–9]. HBV cccDNA plays a crucial role in the HBV life cycle and is a critical obstacle of anti-HBV therapy. As a host cellular nucleosome assembly, HBV cccDNA accumulates in hepatocyte nuclei as a stable and chromatin-like minichromosome organized by histone and non-histone proteins [10]. In host cells, cellular proteins, such as histone H3, H4, H2A, and H2B, are the main structural components assembled onto cccDNA to form the original cccDNA minichromosome [11, 12]. The virally encoded core antigen (HBc) is also a structural component of the HBV cccDNA minichromosome, which preferentially binds to HBV double-stranded DNA [10, 11]. HBV cccDNA minichromosome provides numerous options for dynamic epigenetic control of cccDNA transcriptional activity [13, 14]. In addition, viral protein hepatitis B virus X protein (HBx) and cellular protein p300 bind to cccDNA minichromosome [14].

The epigenetic regulation induced by interferon-α (IFN-α) is the most important mechanism in the modulation of cccDNA minichromosome [15]. IFN-α and lymphotoxin-β receptor activation can promote the induction of mutations in the HBV genome by upregulating APOBEC3A and APOBEC3B, respectively [10, 16]. Recently, our group reported that HBx-elevated MSL2 modulates HBV cccDNA by inducing the degradation of APOBEC3B [17]. It has been reported that a series of enzymes regulate histone methylation of HBV cccDNA minichromosome, including SETDB1, LSD1, Set1A, and PRMT5 [18–20]. In addition, cellular factors, such as CBP, p300, and PCAF, are required for histone acetylation of HBV cccDNA minichromosome and HBV DNA replication [14]. IFN-α represses viral transcription via epigenetic mechanisms and HBV DNA replication involving corepressors and components of polycomb repressive complex 2 (PRC2) [15]. HBV ISRE mediates STAT1 and STAT2 recruitment to cccDNA, which contributes to the cccDNA transcription and replication [15]. IFN-α treatment reduces the binding of STAT1 and STAT2 transcription factors to cccDNA.

Considering the effect of IFN-α on histone acetylation on cccDNA, in response to IFN-α, cccDNA-bound histones become hypoacetylated, and both components of the transcriptional repressor complex PRC2, namely, YY1 and Ezh2, are actively recruited onto cccDNA. Administration of IFN-α results in cccDNA-bound histone hypoacetylation as well as active recruitment of transcriptional corepressors to the cccDNA [15]. It has been reported that cccDNA transcription requires histone deacetylase activity, and IFN-α induces profound and long-lasting suppression of cccDNA transcription, which requires protein synthesis and is related to the reduction of acetylated histone H3 lysine 9 (H3K9) and 27 (H3K27) on the cccDNA minichromosome [15]. However, the role of IFN-α in the modulation of epigenetic regulation of HBV cccDNA minichromosome is poorly understood.

Lysine succinylation is a recently discovered protein posttranslational modification (PTM) in which the modification enzyme transfers the succinyl group from the succinyl donor (succinyl-CoA) to the lysine residue of the protein substrate [21]. Succinylation is widespread among diverse mitochondrial metabolic enzymes and on extramitochondrial cytosolic and nuclear proteins [22]. Lysine succinylation induces more substantial changes to a protein’s chemical properties than lysine methylation and acetylation. The succinylation of the lysine residue changes the charge status from 1 to −1, leading to more significant changes in protein structure and function. Lysine succinylation presents important cellular functions in the regulation of chromatin-based processes [23]. In addition to physiological processes, lysine succinylation is also associated with pathophysiological processes and diseases [23]. Lysine succinylation is closely correlated with fatty acid metabolism and amino acid metabolism [22–24].

Lysine acetyltransferase 2A (KAT2A, also termed GCN5), a member of the GCN5-related N-acetyltransferase (GNAT) superfamily, has been identified as a histone acetyltransferase (HAT) that binds to acetyl-CoA and transfers its acetyl group to histones [25]. In addition, GCN5 functions as a histone succinyltransferase by directly transferring the succinyl group from succinyl-CoA to histone H3 lysine 79 (H3K79), which is important for the regulation of gene expression in tumor cells [25]. GCN5 is recruited to the HBV cccDNA minichromosome, resulting in HBV replication [14]. However, the role of IFN-α in the modulation of histone succinylation in HBV cccDNA minichromosome is elusive [26].

In this study, we are interested in the role of IFN-α in the epigenetic regulation of HBV cccDNA minichromosome. Strikingly, we found that IFN-α conferred epigenetic regulation of cccDNA minichromosome by affecting GCN5-mediated succinylation of histone H3K79 to clear HBV cccDNA, and histone succinylation of HBV cccDNA minichromosome could promote the transcription of HBV cccDNA minichromosome. Our findings provide new insights into the role of lysine succinylation in the biology of HBV cccDNA minichromosome.

## Results

### Histone H3K79 succinylation is anchored on the HBV cccDNA minichromosome

**Fig. 1:**
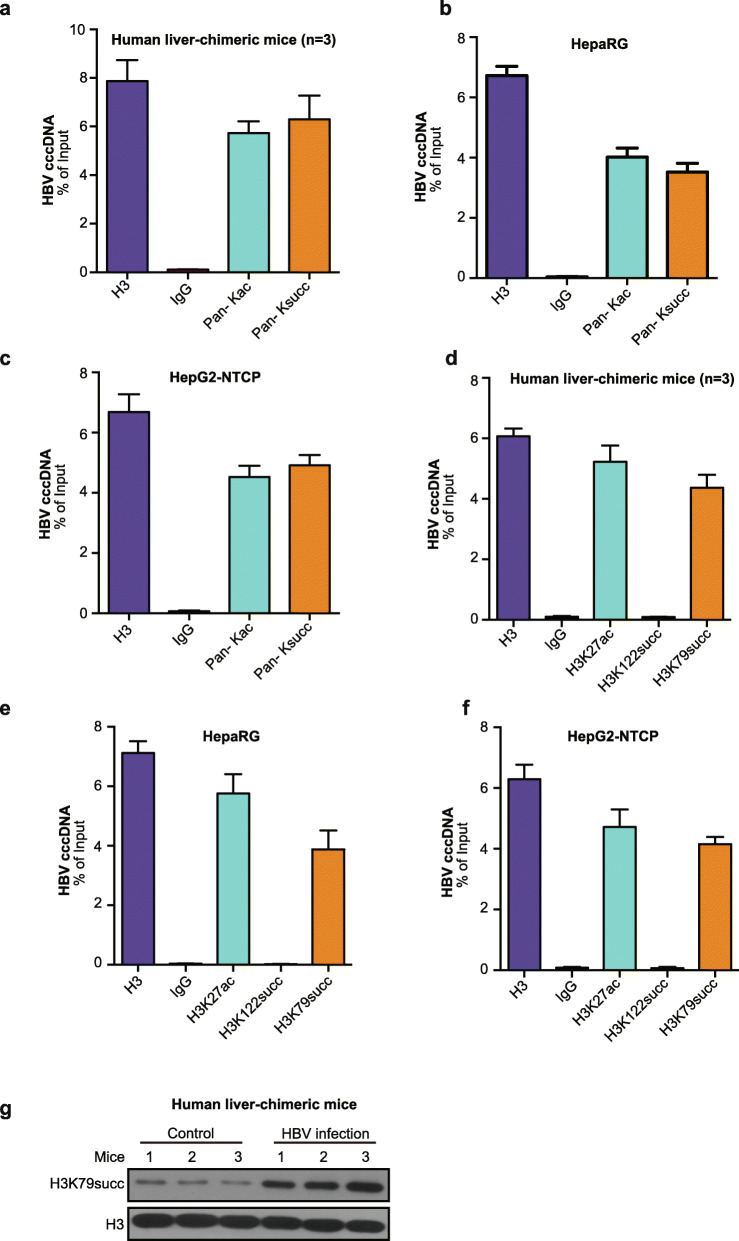
Histone H3K79 succinylation is anchored on the HBV cccDNA minichromosome. **a** The succinylation of the cccDNA minichromosome was examined by ChIP-qPCR in the liver of HBV-infected human liver-chimeric mice (*n* = 3). **b**, **c** HepaRG and HepG2-NTCP cells were infected with HBV at an MOI of 600 vp/cell. The succinylation of the cccDNA minichromosome was determined by ChIP-qPCR in the cells. The mean ± SD of at least three experiments is shown. **d** The succinylation of histone H3K79 in cccDNA minichromosome was analyzed by ChIP-qPCR in livers from HBV-infected human liver-chimeric mice (*n* = 3). **e**, **f** HepaRG and HepG2-NTCP cells were infected with HBV at an MOI of 600 vp/cell. Histone H3K79 succinylation of the cccDNA minichromosome was assessed by ChIP-qPCR in the cells. **g** The levels of histone H3K79 succinylation were examined by Western blot analysis from the livers of human liver-chimeric mice (*n* = 3) and HBV-infected human liver-chimeric mice (*n* = 3). The experiments were repeated at least three times

Similar to the nucleosome in host cells, HBV cccDNA is organized into a chromatin-like minichromosome by histones and non-histone proteins [27]. This minichromosome structure provides multiple PTMs, such as acetylation and methylation, modulating HBV cccDNA and HBV replication [26]. Succinylation is an evolutionarily conserved and widespread PTM similar to acetylation and has been recently identified [21, 28]. However, the role of succinylation in the epigenetic regulation of cccDNA minichromosome has not been reported. Accordingly, we were interested in whether succinylation was involved in the epigenetic regulation of HBV cccDNA minichromosome. Interestingly, we identified succinylation in the HBV-infected human liver-chimeric mouse model [29]. Surprisingly, using the pan-Ksucc antibody, we demonstrated the succinylation of cccDNA minichromosome by cccDNA-ChIP assays in HBV-infected human liver-chimeric mice (Fig. 1a). Moreover, we validated the data in HBV-expressing HepaRG and HepG2-NTCP cells (Fig. 1b and c), suggesting that histone H3 succinylation is anchored on the HBV cccDNA minichromosome.

It has been reported that the succinylation of histone H3K79 is involved in epigenetic regulation [25]. However, whether specific histone succinylation confers epigenetic regulation of the HBV cccDNA minichromosome is unclear. Interestingly, the succinylation of histone H3K79, but not histone H3K122, was observed in the cccDNA minichromosome from HBV-infected human liver-chimeric mice, HepaRG, and HepG2-NTCP cells (Fig. 1d-f), suggesting that histone H3K79 succinylation may be required for the epigenetic regulation of cccDNA minichromosome. In addition, acetylation and histone H3K27 acetylation were used as positive controls in the system. In addition, Western blot analysis revealed that the levels of histone H3K79 succinylation were significantly elevated in HBV-infected human liver-chimeric mice (Fig. 1g), suggesting that the succinylation of histone H3K79 may be involved in HBV replication. Thus, we conclude that histone H3K79 succinylation is anchored on the HBV cccDNA minichromosome.

### Succinylation of histone H3K79 contributes to the transcription of HBV DNA

**Fig. 2:**
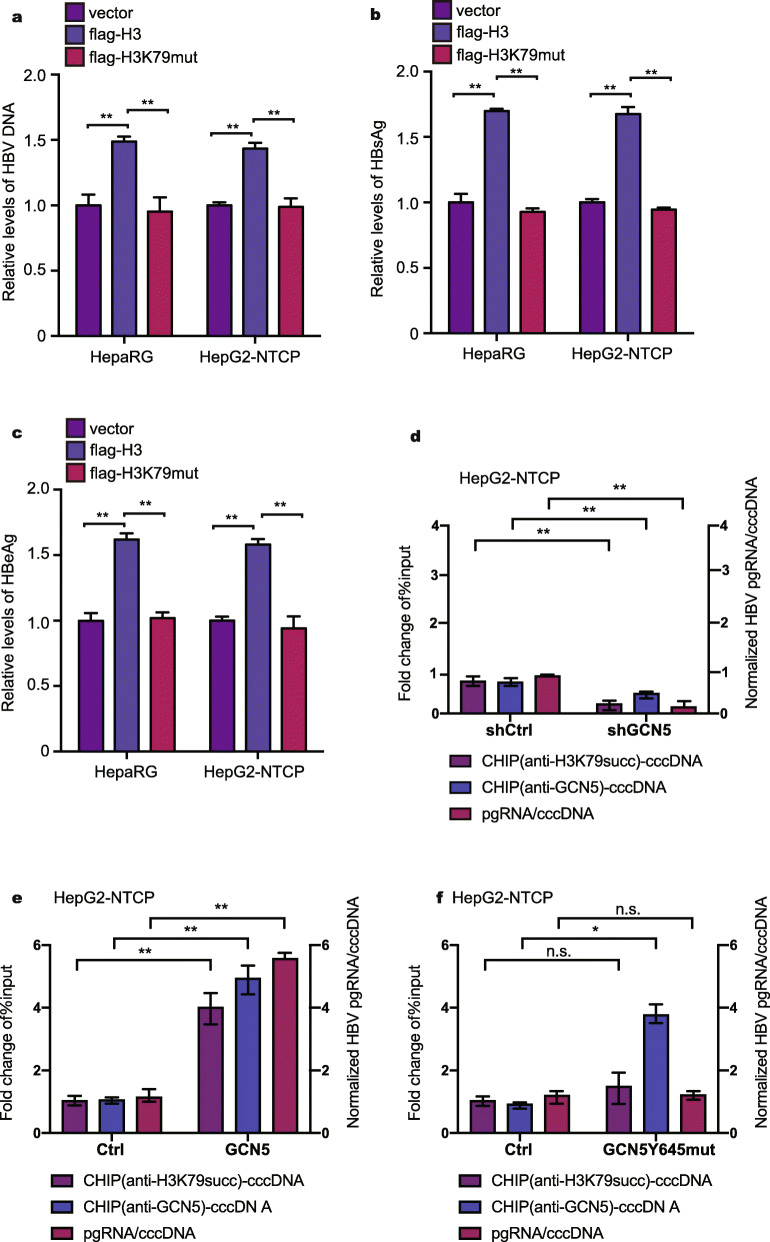
Succinylation of histone H3K79 contributes to the transcription of HBV DNA. **a**-**c** The levels of HBV DNA, HBsAg, and HBeAg in the supernatant of medium were analyzed by qPCR and ELISA assays in HBV-infected HepG2-NTCP cells continuously transfected with flag-vector/flag-H3/flag-H3K79mut at −4, 0, 4, and 8 days after infection (dpi), respectively. **d**-**f** shGCN5 was transduced into HBV-infected HepG2-NTCP cells in 100-mm dishes for 5 days. GCN5 or GCN5Y645mut was transduced into HBV-infected HepG2-NTCP cells in 100-mm dishes for 6 days. The cells were harvested and used for ChIP assays with the indicated antibodies. Levels of the specific proteins on HBV cccDNA were analyzed by ChIP-qPCR. The mean ± SD of at least three experiments is shown. *P < 0.05, **P < 0.01. Abbreviation: N.S., not significant

Generally, the modification of histones plays critical roles in epigenetic regulation and chromatin remodeling, widely affecting gene expression [30–34]. Multiple studies have demonstrated that epigenetic modifications of HBV cccDNA, such as histone modifications and DNA methylation, participate in the regulation of the transcriptional activity of HBV cccDNA [35]. However, whether the succinylation of histone H3K79 is involved in the transcription of HBV DNA remains unknown. Interestingly, we observed that the overexpression of histone H3 could increase the levels of HBV DNA, hepatitis B surface antigen (HBsAg), and hepatitis B e antigen (HBeAg) in the supernatant from HBV-expressing HepaRG and HepG2-NTCP cells, whereas the mutant of histone H3K79 lysine (K) to arginine (R) failed to work in the system (Fig. 2a-c, Fig. S1a), suggesting that the PTM of histone H3K79 can modulate HBV replication. Because the site histone H3K79 lysine (K) can be regulated by PTMs other than succinylation [36], to rule out other PTMs, we needed to distinguish the effect of the mutant of histone H3K79 lysine (K) to arginine (R) on unique succinylation. Because GCN5Y645 is the specific site for succinylation, we generated a mutant of GCN5Y645 to A [25]. Accordingly, our data showed that the knockdown of GCN5 reduced the binding of GCN5 to cccDNA and the level of H3K79 succinylation and decreased cccDNA transcription activity in HepG2-NTCP and HepaRG cells (Fig. 2d, Fig. S1c). The efficiency of knockdown and overexpression of GCN5 were validated by Western blot analysis in 293 T cells (Fig. S1b). The overexpression of GCN5 led to the opposite results in the cells (Fig. 2e, Fig. S1d). Importantly, the GCN5 mutation (Y645A) increased the binding of GCN5 to cccDNA but failed to affect the levels of H3K79 succinylation on cccDNA and the cccDNA transcription activity in the cells (Fig. 2f, Fig. S1e), suggesting that GCN5 can modulate the replication of cccDNA through H3K79 succinylation on cccDNA, rather than other PTMs. Thus, we conclude that the succinylation of histone H3K79 contributes to the transcription of HBV DNA.

### GCN5 is responsible for the succinylation of histone H3K79 on HBV cccDNA minichromosome

**Fig. 3:**
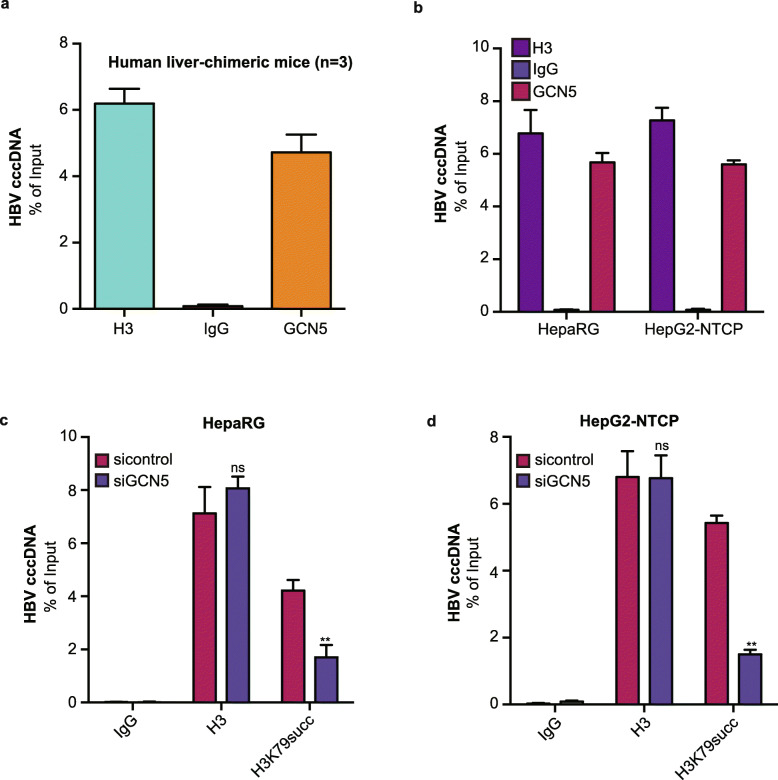
GCN5 is responsible for the succinylation of histone H3K79 on the HBV cccDNA minichromosome. **a** The interaction of GCN5 with cccDNA was measured by ChIP-qPCR from the livers from HBV-infected human liver-chimeric mice (*n* = 3). **b** HepaRG and HepG2-NTCP cells were infected with HBV at an MOI of 600 vp/cell. The deposition of GCN5 on cccDNA was measured by ChIP-qPCR in HBV-infected HepaRG and HepG2-NTCP cells. **c**, **d** HepaRG and HepG2-NTCP cells were infected with HBV at an MOI of 600 vp/cell and continuously transfected with siGCN5#1 (100 nM) at −4, 0, and 4 dpi. The succinylation of histone H3K79 on cccDNA minichromosome was verified by ChIP-qPCR 8 dpi in the cells. The mean ± SD of at least three experiments is shown. Statistically significant differences are indicated as follows: **P < 0.01; ns, no significance

It has been reported that GCN5, as a histone succinyltransferase, is responsible for the succinylation of histone H3K79 to regulate gene expression [25]. GCN5 can be recruited to the HBV cccDNA minichromosome to enhance HBV replication [14]. Accordingly, we hypothesized that GCN5 might modulate the succinylation of histone H3K79 on cccDNA minichromosome. As expected, cccDNA-ChIP assays validated that GCN5 bound to cccDNA minichromosome in HBV-infected human liver-chimeric mice and HBV-expressing HepaRG and HepG2-NTCP cells (Fig. 3a and b), supporting that GCN5 contributes to the epigenetic regulation of HBV cccDNA minichromosome. Then, siGCN5 #1 was selected from two GCN5 siRNAs in 293 T cells. Its efficiency was verified, and it was used in HepaRG and HepG2-NTCP cells (Fig. S2). Interestingly, cccDNA-ChIP assays showed that siGCN5 significantly inhibited the level of succinylation of histone H3K79 on cccDNA minichromosome in the above cells (Fig. 3d and e). We conclude that GCN5 is responsible for the succinylation of histone H3K79 on the cccDNA minichromosome.

### GCN5 positively regulates HBV replication in the liver

**Fig. 4:**
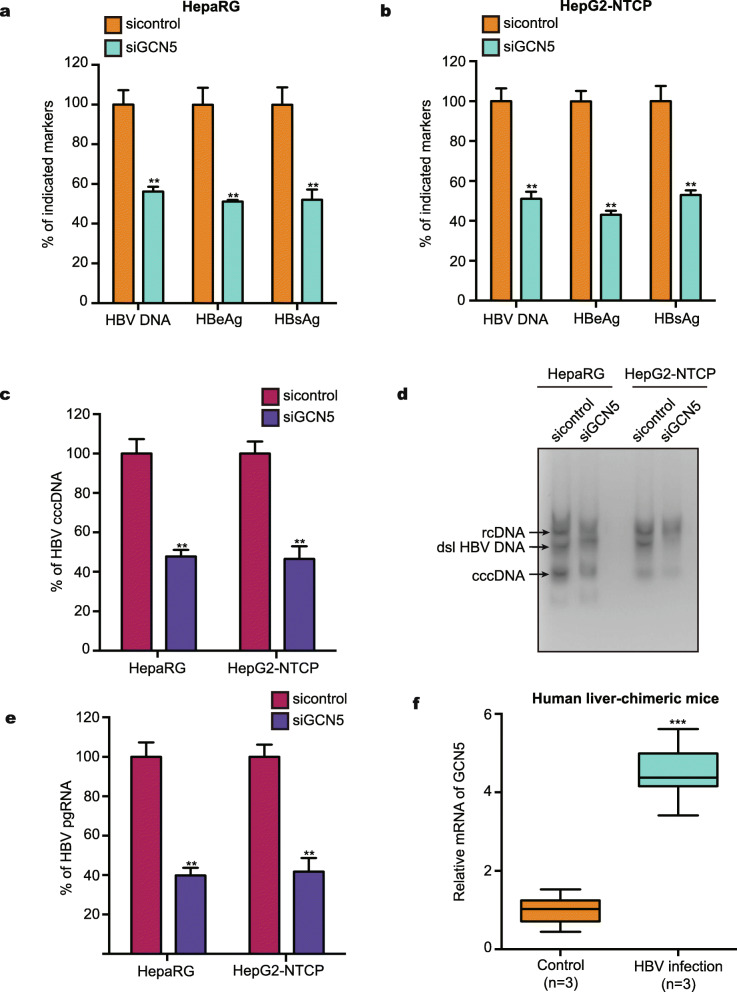
GCN5 positively regulates HBV replication in the liver. **a** The levels of HBV DNA, HBeAg, and HBsAg in the supernatant of the medium were measured by qPCR and ELISA in the cells. **b** The levels of HBV cccDNA were analyzed by selective qPCR 8 dpi in the cells. **c** HepG2-NTCP and dHepaRG cells were infected with HBV at an MOI of 600 vp/cell and continuously transfected with siGCN5#1 (100 nM) at −4, 0, and 4 dpi. The levels of HBV DNA, HBeAg, and HBsAg in the supernatant of the medium were measured by qPCR and ELISA in the cells. **d** The levels of HBV cccDNA were analyzed by Southern blot analysis in the cells. **e** HepG2-NTCP cells and HepaRG cells were infected with HBV at an MOI of 600 vp/cell and continuously transfected with siGCN5#1 (100 nM) at 8 dpi. RT-qPCR analysis was used to quantify the levels of pgRNA. **f** The mRNA levels of GCN5 were examined by RT-qPCR in the liver from human liver-chimeric mice (*n* = 3) and HBV-infected human liver-chimeric mice (*n* = 3). The mean ± SD of at least three experiments is shown. Statistically significant differences are indicated as follows: **P < 0.01; ***P < 0.001

It has been reported that GCN5 can be recruited to the HBV cccDNA minichromosome to enhance HBV replication [14]. In our study, we verified that siGCN5 reduced the levels of HBV DNA, HBsAg, HBeAg, cccDNA, and pgRNA in HepaRG and HepG2-NTCP cells (Fig. 4a-e), supporting that GCN5 promotes HBV replication and cccDNA accumulation. We further evaluated the relationships between GCN5 and HBV cccDNA in clinical liver cancer tissues and paracancerous liver tissues. Our data found that 39 samples were positive for HBV DNA in 43 paracancerous liver tissues, of which 15 samples were positive for HBV cccDNA. Interestingly, the expression levels of GCN5 were remarkably elevated in HBV cccDNA-positive tissues (Fig. S3). Importantly, we observed that the GCN5 level was elevated in the liver tissues of HBV-infected human liver-chimeric mice (*n* = 3), supporting that GCN5 is closely related to HBV infection (Fig. 4f). Thus, we conclude that GCN5 contributes to the replication of HBV in the liver.

### IFN-α inhibits the succinylation of histones on HBV cccDNA minichromosome

**Fig. 5:**
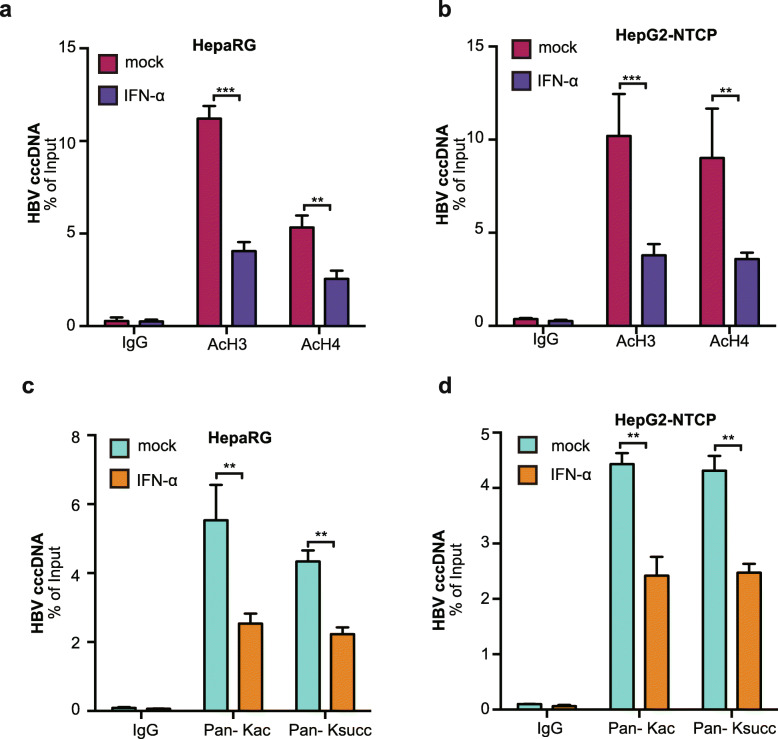
IFN-α inhibits the succinylation of histones on HBV cccDNA minichromosome. **a**, **b** HepaRG cells were infected with HBV at an MOI of 600 vp/cell and treated with IFN-α for 3 days at the indicated dose. **a** The acetylation of histones on cccDNA was determined by ChIP-qPCR 8 dpi in HepaRG cells. **b** The acetylation of histones on cccDNA was determined by ChIP-qPCR 8 dpi in HepG2-NTCP cells. **c** The acetylation and succinylation of cccDNA were analyzed by ChIP-qPCR 8 dpi in the cells using pan-Kac and pan-Ksucc antibodies. **d** The acetylation and succinylation of cccDNA were analyzed by ChIP-qPCR 8 dpi in the cells using pan-Kac and pan-Ksucc antibodies. The mean ± SD of at least three experiments is shown. Statistically significant differences are indicated as follows: **P < 0.01; ***P < 0.001

IFN-α is an efficient agent for inhibition of HBV cccDNA clinically, and IFN-α represses viral transcription via epigenetic mechanisms involving chromatin remodeling polycomb repressive complex 2 (PRC2), which can inhibit histone acetylation on HBV cccDNA minichromosome [15]. Accordingly, we were interested in whether IFN-α affected the succinylation of histones on the cccDNA minichromosome. cccDNA-ChIP assays verified that IFN-α reduced the levels of acetylation of histone H3 and H4 on HBV cccDNA minichromosome in HBV-infected HepaRG and HepG2-NTCP cells as controls (Fig. 5a and b). Strikingly, we further validated that IFN-α inhibited the succinylation of HBV cccDNA minichromosome in the cells (Fig. 5c and d). Therefore, we conclude that IFN-α is able to depress the succinylation of histones on HBV cccDNA minichromosome.

### IFN-α depresses the succinylation of histone H3K79 mediated by GCN5 on HBV cccDNA minichromosome

**Fig. 6:**
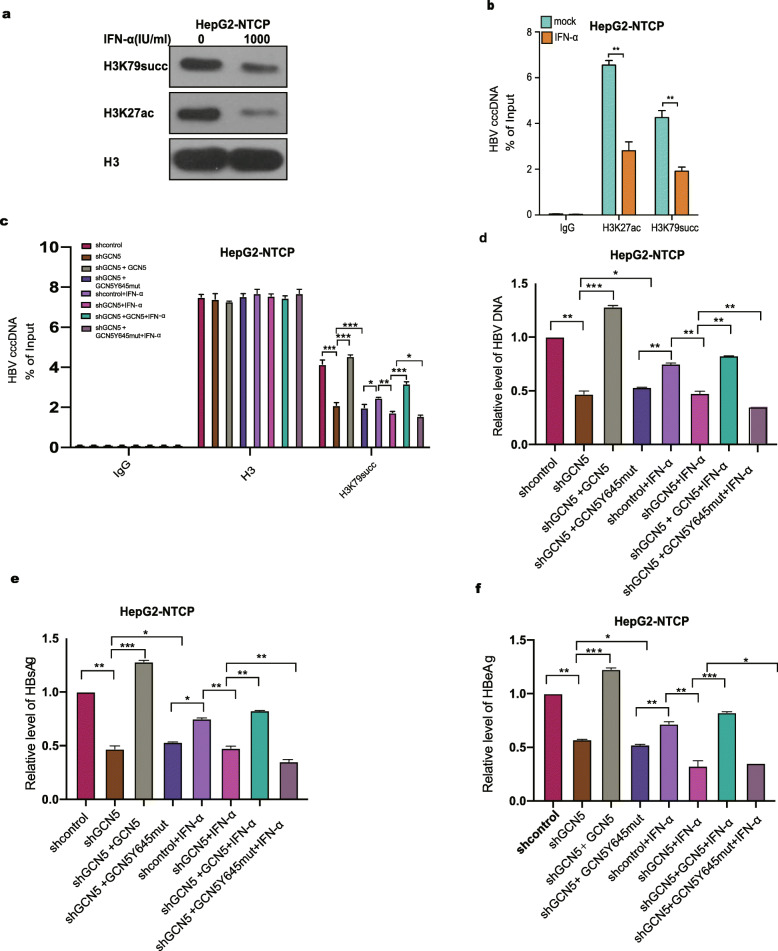
IFN-α depresses the succinylation of histone H3K79 on HBV cccDNA minichromosome. **a** Hepe-NTCP cells were infected with HBV at an MOI of 600 vp/cell and treated with IFN-α for 3 days at the indicated dose. The levels of histone H3K27 acetylation and histone H3K79 succinylation were measured by Western blot analysis in the cells. **b** Histone H3K27 acetylation and histone H3K79 succinylation on cccDNA were assessed by ChIP-qPCR in the cells. **c** HepG2-NTCP cells with depleted endogenous GCN5 and reconstituted expression of wild-type flag-GCN5 or flag-GCN5Y645mut were infected with HBV at an MOI of 600 vp/cell and treated with IFN-α for 3 days at the indicated dose. Histone H3K79 succinylation on cccDNA was assessed by ChIP-qPCR in the cells. **d**, **e**, **f** The levels of HBV DNA, HBsAg, and HBeAg were analyzed by qPCR and ELISA assays in the supernatant of the medium from HepG2-NTCP cells. The mean ± SD of at least three experiments is shown. Statistically significant differences are indicated as follows: **P < 0.01; ***P < 0.001

Next, we identified the effect of IFN-α on histone H3K79 on HBV cccDNA minichromosome. Surprisingly, Western blot analysis and a ChIP assay showed that IFN-α reduced the levels of succinylation of histone H3K79 on HBV cccDNA minichromosome and levels of cccDNA in HBV-infected and HepG2-NTCP cells (Fig. 6a, b), and the acetylation of histone H3K27 was used as a positive control. Moreover, Western blot analysis and a ChIP assay revealed that treatment with IFN-α remarkably decreased the levels of histone H3K79 succinylation on HBV cccDNA minichromosome and the levels of cccDNA in HepaRG cells (Fig. S4a and b). To further validate this finding, we further examined the effect of IFN-α on histone H3K79 succinylation combined with GCN5 in the system. We depleted endogenous GCN5 by shRNA in HepG2-NTCP and HepaRG cells and then reconstituted the expression of wild-type flag-GCN5 or flag-GCN5Y645mut in the cells. As expected, our data showed that IFN-α treatment significantly affected the levels of HBV cccDNA, HBV DNA, HBsAg, and HBeAg in HepG2-NTCP and HepaRG cells in different situations, such as knockdown or overexpression of GCN5 or ectopic overexpression of the GCN5Y645 mutant (Fig. 6c-f and Fig. S4c-f), suggesting that IFN-α represses the succinylation of histone H3K79 mediated by GCN5 on HBV cccDNA minichromosome. Thus, we conclude that IFN-α confers epigenetic regulation of the HBV cccDNA minichromosome by modulating GCN5-mediated succinylation of histone H3K79 to clear HBV cccDNA.

## Discussion

Chronic HBV infection remains a significant health problem worldwide and is a key risk factor for the development of liver cancer [4]. As a direct template for HBV replication, HBV cccDNA minichromosome plays critical roles in the viral persistence [26]. Accumulated evidence demonstrates that the cccDNA transcription is regulated by epigenetic machinery, including histone acetylation and methylation [13]. Lysine succinylation is a frequently occurring modification in prokaryotes and eukaryotes, and histone succinylation contributes to the cancer epigenetics [28]. However, unlike multiple epigenetic modifications that are well documented, novel histone modifications such as histone succinylation on HBV cccDNA minichromosome remain unclear. It has been reported that IFN-α represses the viral transcription via epigenetic mechanisms involving chromatin remodeling PRC2. Moreover, IFN-α treatment reduces the binding of STAT1 and STAT2 transcription factors to cccDNA to repress viral transcription. IFN-α can result in the hypoacetylation of cccDNA-bound histones and the recruitment of transcriptional corepressors to cccDNA [15]. In this study, we were interested in the role of IFN-α in the modulation of epigenetic regulation of HBV cccDNA minichromosome.

We tried to identify the role of histone succinylation in the epigenetic regulation of HBV cccDNA minichromosome. Similarly to host nucleosome assembly, nuclear cccDNA exists in hepatocyte nuclei as a stable minichromosome organized by histone and non-histone viral and cellular proteins [12]. In recent years, a number of epigenetic markers of HBV cccDNA minichromosome associated with viral transcription have been identified [13, 18, 26]. The role of methylation and acetylation of cccDNA-bound histones in the regulation of HBV transcription has been well recognized [20]. Recently, the succinylation of lysine has been identified, playing crucial roles in the processes of physiology and pathology [21, 28]. Hence, we are interested in the significance of histone succinylation in the epigenetic regulation of HBV cccDNA minichromosome. Surprisingly, we observed the succinylation of histones and further identified the succinylation of histone H3K79 on HBV cccDNA minichromosome. Our data demonstrated that the levels of histone H3K79 succinylation were significantly elevated in the livers of HBV-infected human liver-chimeric mice. It suggests that histone H3K79 succinylation plays an important role in the epigenetic regulation of the HBV cccDNA minichromosome. Functionally, our data showed that the overexpression of histone H3 could increase the levels of HBV DNA, HBsAg, and HBeAg, whereas the histone H3K79 mutant (lysine K to arginine R) failed to work. It suggests that the succinylation of histone H3K79 might promote HBV replication. Hence, we first report that histone H3K79 succinylation is involved in the epigenetic regulation of HBV cccDNA minichromosome in addition to histone acetylation and methylation. The use of anti–acetyl-H4/-H3 specific antibodies to immunoprecipitate transcriptionally active chromatin revealed that HBV replication was regulated by the acetylation status of cccDNA-bound H3/H4 histones [37]. However, the role of succinylation compared to acetylation in the modulation of HBV cccDNA minichromosome is not clear.

Given that GCN5 is a histone succinyltransferase responsible for the succinylation of histone H3K79 to regulate gene expression [25] and that GCN5 is recruited to the HBV cccDNA minichromosome, leading to HBV replication [14], we hypothesized that GCN5 might modulate the succinylation of histone H3K79 on the HBV cccDNA minichromosome to enhance HBV replication. As expected, we validated that GCN5 could bind to the HBV cccDNA minichromosome. This implies that GCN5 may be involved in the modulation of histone H3K79 on HBV cccDNA minichromosome. Functionally, we found that siGCN5 significantly inhibited HBV replication, HBV cccDNA accumulation, and the level of histone H3K79 succinylation on the cccDNA minichromosome. It suggests that GCN5 is responsible for the modulation of histone H3K79 succinylation on cccDNA minichromosome, leading to HBV replication and HBV cccDNA accumulation.

It has been reported that IFN-α represses viral transcription via epigenetic mechanisms involving histone acetylation [15]. In this study, our data showed that IFN-α reduced the levels of succinylation of histone H3K79 and acetylation of histone H3K27 in the cells. We validated that in vitro treatment with IFN-α could reduce the acetylation of histone H3 and H4 on cccDNA minichromosome as a control. Moreover, we found that IFN-α inhibited histone H3K79 succinylation in the HBV cccDNA minichromosome. Thus, we conclude that GCN5-mediated histone H3K79 succinylation contributes to the epigenetic regulation of the HBV cccDNA minichromosome, leading to HBV replication.

**Fig. 7:**
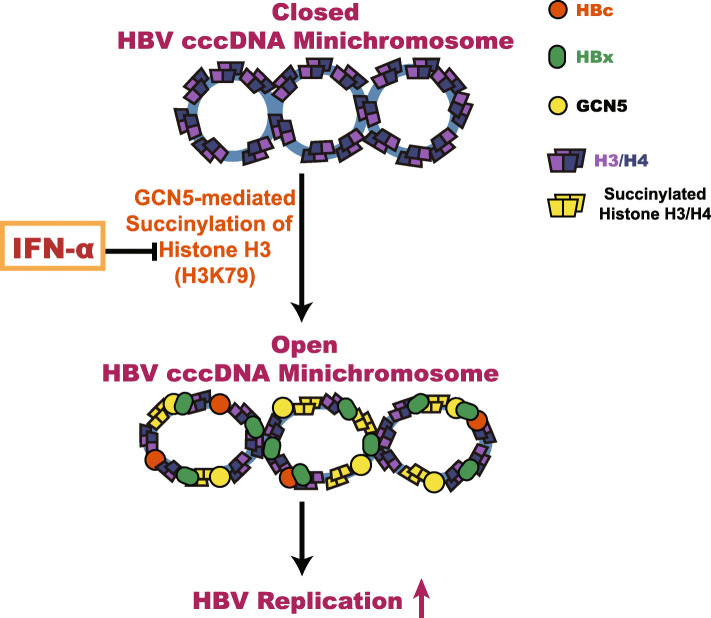
A model of histone H3K79 succinylation modulating the epigenetic regulation of HBV cccDNA minichromosome. In this model, histone H3K79 succinylation is identified, which contributes to the epigenetic regulation of the HBV cccDNA minichromosome, leading to HBV replication. GCN5 is responsible for histone H3K79 succinylation of the HBV cccDNA minichromosome. IFN-α is able to inhibit histone H3K79 succinylation of HBV cccDNA minichromosome

We summarize a model for the role of histone succinylation mediated by GCN5 in epigenetic regulation of the HBV cccDNA minichromosome (Fig. 7). Notably, IFN-α confers epigenetic regulation of the HBV cccDNA minichromosome by modulating GCN5-mediated succinylation of histone H3K79 to clear HBV cccDNA. Our findings provide new insights into the mechanism by which IFN-α modulates the epigenetic regulation of HBV cccDNA minichromosome.

## Conclusions

In conclusion, our data reveal that IFN-α confers epigenetic regulation of HBV cccDNA minichromosome by modulating GCN5-mediated succinylation of histone H3K79 to clear HBV cccDNA. Our findings provide new insights into the mechanism by which IFN-α modulate the epigenetic regulation of HBV cccDNA minichromosome.

## Materials and methods

### Generation of human liver-chimeric mice

Human liver-chimeric mice were generated by VITALSTAR (Beijing, China) [29]. Primary human hepatocytes (PHH) were transplanted into 3-week-old urokinase-type plasminogen activator/severe combined immunodeficient beige (uPA/SCID-bg) mice (male and female) by intrasplenic injection as described [10, 38]. Engraftment and viability of PHHs were assessed by quantification of human serum albumin by enzyme-linked immunosorbent assay (Human Albumin ELISA Kit, Immunology Consultants Lab, Portland, USA). Then, the uPA/SCID-bg mice were infected with 2.5 × 10^8^ IU/ml (0.2 ml/mouse) HBV particles from the supernatant of HepAD38 cells (tet-off) and sacrificed 8 weeks after virus inoculation. Serum HBV load in the mice was determined by quantitative PCR (Da An Gene, Guangzhou, China) before sacrifice. The Institute Research Ethics Committee at Nankai University approved the study protocol.

### HBV inocula, cell cultures, and HBV infection

HBV inocula were prepared as previously described [10, 17]. Briefly, media from HepAD38 cells at days 7-15 postinduction of HBV by depletion of tetracycline were recovered every 3 days. Media were filtered through a 0.45-μm filter and precipitated with 10% PEG 8000 and 2.3% NaCl. The precipitates were washed and resuspended in the medium at 200-fold concentration. HBV DNA was quantified by real-time PCR. Primary human hepatocytes (PHHs) were purchased from RIDL (Shanghai, China), cultured, and infected with HBV as described [39, 40]. Briefly, PHH was maintained in William’s E medium (Gibco-Invitrogen) with 5% FCS, 7 × 10^−5^ M hydrocortisone hemisuccinate, 5 μg/ml insulin and 2% DMSO (Sigma-Cell Culture reagent). HepaRG differentiation (dHepaRG) has been described previously [41]. Briefly, cells were maintained for 2 weeks in standard medium and then for at least 2 weeks in standard medium with 1.8% DMSO and EGF (5 ng/ml) (PeproTech-Tebu, France). An expression plasmid for hNTCP was transfected into HepG2 cells with TransIT-LT1 (Mirus, USA) according to the manufacturer’s instructions to establish HepG2-NTCP cells [1, 42]. HBV infections were also performed as described [10, 16, 18]. Briefly, HBV infection was carried out with HBV purified from the supernatant of HepAD38 (tet-off) cells by heparin affinity chromatography and subsequent concentration via sucrose gradient ultracentrifugation at an MOI of 600 vp/cell viral titer in PHH, HepaRG, and HepG2-NTCP cells. Infection was performed by using 5% PEG 8000 and William’s E medium containing 10% FBS, penicillin/streptomycin, human insulin (350 μl, Sigma I9278, USA), hydrocortisone (5 μg/ml, Sigma H2270), and 1.8% DMSO (Sigma 2650). Transfection was performed using Lipofectamine RNAiMAX (Thermo Fisher Scientific, USA) and Lipofectamine MessengerMAX (Thermo Fisher Scientific, USA) according to the manufacturer’s protocol in the PHH, dHepaRG, and HepG2-NTCP cell lines. IFN-α (Roferon-A, Roche) was used at 1000 IU/ml unless otherwise indicated.

### HBV cccDNA-ChIP

ChIP experiments were carried out in infected cells for 8 days postinfection as described with minor modifications [18]. Briefly, cells were fixed with 1% formaldehyde for 10 min at 37 °C and quenched with 0.125 M glycine. For nuclear extract preparation, cells were lysed in buffer A (0.25% Triton X-100, 10 mM Tris pH 8.0, 0.5 mM pefablock, EDTA-free protease inhibitors, Roche). After centrifugation, nuclei were washed in buffer B (0.2 M NaCl, 10 mM Tris pH 8.0, 0.5 mM pefablock, EDTA-free protease inhibitors), centrifuged and lysed in nuclei lysis buffer (1% SDS, 10 mM EDTA, 50 mM Tris pH 8.0, 0.5 mM pefablock, EDTA-free protease inhibitors). After sonication, lysates were diluted 1:10 with 0.01% SDS, 1% Triton X-100, 1.2 mM EDTA, 16.7 mM Tris pH 8.0, 167 mM NaCl, 0.5 mM pefa-block and EDTA-free protease inhibitors. Chromatin was then subjected to overnight immunoprecipitation at 4 °C using 2-5 μg of antibodies listed in Table S1. Negative controls with nonspecific immunoglobulin (Millipore PP64B) were included in each experiment. Immune complexes were incubated with protein A/G agarose beads at 4 °C, washed, and eluted in 1% SDS and 0.1% NaHCO_3_. Immunoprecipitated DNA was quantified by qPCR using cccDNA-specific primers. Samples were normalized to input DNA using the ∆Ct method and were ∆Ct = Ct (input)−Ct (immunoprecipitation) and calculated as a percentage of the input. The results are expressed as the average of at least three independent experiments. The standard error of the mean is indicated. Statistical differences were analyzed by Student’s *t* test.

### Patient samples

Forty-three paracancerous liver tissues from HCC patients utilized in this study were immediately obtained from Tianjin First Center Hospital (Tianjin, P.R. China) and Tianjin Medical University Cancer Institute and Hospital (Tianjin, P.R. China) after surgical resection. Clinicopathological information about the patients was obtained from patient records and is summarized in Table S2. Written consent for the use of their tissues for research purposes after the operation was obtained from patients. The Institute Research Ethics Committee at Nankai University approved the study protocol.

### RNA extraction, reverse transcription-polymerase chain reaction (RT-PCR), and quantitative real-time PCR (RT-qPCR)

Total RNA was extracted from the liver tissues from human liver-chimeric mice (or liver tissues from patients) using TRIzol reagent (Invitrogen, Carlsbad, CA, USA). First-strand cDNA was synthesized as previously reported. RT-qPCR was performed using a Bio-Rad sequence detection system according to the manufacturer’s instructions using the double-stranded DNA-specific SYBR GreenPremix Ex TaqTM II Kit (TaKaRa, Ohtsu, Japan). Experiments were conducted in duplicate in three independent assays. Relative transcriptional folds were calculated as 2^−∆∆Ct^. GAPDH was used as an internal control for normalization. The expression levels of GCN5 were analyzed by RT-qPCR in liver tissues of human liver-chimeric mice and HBV-infected human liver-chimeric mice. All the primers used are listed in Table S3.

### Western blot analysis

Total protein lysates were extracted from hepatoma cells with RIPA buffer. Protein concentrations were measured using the Bradford assay, and 20-50 μg protein extracts were subjected to SDS-PAGE. Then, the proteins were transferred to a nitrocellulose membrane, blocked with 5% nonfat milk, and incubated with primary antibodies for 1 h at room temperature (RT). After incubation with secondary antibody against mouse (1:10,000) or rabbit (1:10,000) for 1 h at 37 °C, the membrane was visualized using the ECL Western Blotting Detection Kit (GE Healthcare, Waukesha, WI, USA). The succinylation levels of histone H3K79 were measured by Western blot analysis in liver tissues of the mice. The Institute Research Ethics Committee at Nankai University approved the study protocol. All antibodies used are listed in Table S1.

### Analysis of HBV replication, HBsAg, and HBeAg

The secretion of HBsAg into the supernatants of cultured cells was measured using a diagnostic kit according to the manufacturer’s instructions (Kehua Bioengineering, Shanghai, China). The cutoff value (COV) for HBsAg analysis was indicated as COV = OD (negative control)/0.100. HBeAg in the supernatants of cultured cells was measured by a diagnostic kit according to the manufacturer’s instructions (Kehua Bioengineering, Shanghai, China). The cutoff value for HBeAg analysis was indicated as follows: COV = OD (negative control) × 2.1 (0 < ODNC ≤ 0.05, COV = 0.05 × 2.1 = 0.105; 0.05 < ODNC ≤ 0.1; ODNC > 0.1, invalidation). qPCR was used to quantify HBV DNA copies in the supernatants of cultured cells according to the manufacturer’s instructions for an HBV DNA quantification kit (Da An Gene, Guangzhou, China) on a Bio-Rad sequence detection system. Total RNA was extracted using TRIzol reagent and reverse transcribed using ReverTra Ace qPCR RT Master Mix from the gDNA remover kit followed by quantitative PCR using Thunderbird SYBR qPCR Mix (both from Toyobo, Japan). The pgRNA primers can also amplify HBV preC RNA [20]. The quantification of HBV pgRNA was normalized to 18 s rRNA [20]. The primers used are listed in Table S3.

### HBV cccDNA isolation

Selective extraction of HBV cccDNA from the cells was achieved by a modified Hirt method as previously described [16, 18, 20]. The cells were lysed in lysis buffer A (50 mM Tris-HCl pH 7.4, 1 mM EDTA, 1% NP-40) containing complete protease inhibitor cocktail for 30 min on ice. After centrifugation, the pelleted nuclei were resuspended in lysis buffer B (10 mM Tris-HCl, 10 mM EDTA, 150 mM NaCl, 0.5% SDS, Proteinase K 0.5 mg/ml) and incubated overnight at 37 °C. Nucleic acids were purified by phenol-chloroform (1:1) extraction and ethanol precipitation.

### HBV cccDNA quantification

Before lysis, the cells were counted using the Cell Counting Chamber Set (Qiujing, Shanghai, China). The procedure of the isolation of HBV cccDNA from the cells has been reported. Quantification of HBV cccDNA was performed by using the method described with minor modifications [16]. Briefly, aliquots of DNA extracted from cell pellets were treated for 1 h at 37 °C with 10 U plasmid-safe ATP-dependent DNase (Epicentre, Madison, WI, USA). qPCR experiments were performed in a Mastercycler ep realplex (Eppendorf, Germany) using a 20 μL reaction volume. Primers used to amplify the cccDNA are listed in Table S3.

### Southern blot analysis of HBV cccDNA

For the detection of cccDNA by Southern blot, the extracted HBV cccDNA sample was subjected to 1.2% agarose gel electrophoresis and transferred onto an Amersham Hybond-N+ membrane (GE Healthcare). The Hybond-N+ membrane was cross-linked in a UV crosslinker chamber with a UV energy dose of 1500 mJ and then probed with “DIG-labelled probes of linear HBx DNA fragments” for 24 h at 37 °C. The membrane was blocked and incubated with anti-digoxigenin-AP (dilute anti-digoxigenin-AP 1:10 000 (75 mU/ml) in blocking solution) overnight at 4 °C. After washing for 15 min, the membrane was placed DNA side up onto a development folder (or hybridization bag), and 1 ml of ready-to-use CSPD (bottle 5) was added. The membrane was immediately covered with the second sheet of the folder to spread the substrate evenly. It was incubated for 5 min at 15-25 °C. When indicated, isolated DNA was digested with EcoRI to linearize the cccDNA.

### siRNA and antibodies

The siRNAs used in this study were purchased from RiboBio (Guangzhou, China). The oligonucleotide sequences of GCN5 siRNA are shown: siGCN5 #1, 5′-CGTGCTGTCACCTCGAATGA-3′; siGCN5 #2, 5′-GCATTAAAGCAGCGTATC-3′. The antibodies used in this study are listed in Table S1.

### Plasmid construct and mutagenesis

The CDS regions of H3 and K79 mutant (K to R) H3 were synthesized and constructed into the plasmid of pCMV-3Tag-1A by Genscript (Nanjing, P.R. China). This constructed plasmid was named flag-H3 and flag-H3K79mut. The CDS regions of H3 and K79 mutant (K to R) H3 were synthesized and cloned into the plasmid of pCMV-3Tag-1A by Genscript (Nanjing, P.R. China). This constructed plasmid was named flag-H3 and flag-H3K79mut. The CDS regions of GCN5 and Y645 mutant (Y to A) GCN5 were synthesized and cloned into the plasmid of pCMV-3Tag-1A by Genscript (Nanjing, P.R. China). This constructed plasmid was named flag-GCN5 and flag-GCN5Y645mut. A control vector was generated by using the control oligonucleotide GCTTCTAACACCGGAGGTCTT. pGIPZ GCN5 shRNA was generated with GCATTAAAGCAGCGTATC. A control pLKO-1 vector was generated with the control oligonucleotide CCGCAGGTATGCACGCGT. shRNAs were obtained from Sigma-Aldrich.

### Statistical analysis

Each experiment was repeated at least three times. Statistical significance was assessed by comparing mean values (± SD) using Student’s *t* test for independent groups and was assumed for *P < 0.05; **P < 0.01; ***P < 0.001.

## Supplementary information

**Additional file 1: Supplementary information. Table S1.** List of antibodies used in this paper. **Table S2.** The characteristics of paracancerous liver tissues of HCC patients. **Table S3.** List of primers used in this paper. **Fig S1**. Succinylation of histone H3K79 contributes to the transcription of HBV DNA. (a) The efficiency of H3 overexpression was validated by Western blot analysis in HepaRG and HepG2 cells. (b) The efficiency of knockdown and overexpression of GCN5 was validated by Western blot analysis in HepaRG and HepG2 cells. (c-e) shGCN5 was transduced into HBV-infected HepaRG cells in 100-mm dishes for 5 days. GCN5 or GCN5Y645mut was transduced into HBV-infected HepaRG cells in 100-mm dishes for 6 days. The cells were harvested and used for ChIP assays with the indicated antibodies. Levels of the specific proteins on HBV cccDNA were analysed by ChIP-qPCR. The mean ± SD of at least three experiments is shown. *P < 0.05, **P < 0.01. Abbreviation: N.S., not significant. **Fig S2**. GCN5 is responsible for the succinylation of histone H3K79 on HBV cccDNA minichromosome. The efficiency of siGCN5 was validated by Western blot analysis in 293T, HepaRG and HepG2 cells. **Fig S3.** Relative mRNA levels of GCN5 were detected by RT-qPCR in cccDNA-positive paracancerous liver tissues (n=15) and cccDNA-negative paracancerous liver tissues (n=15). *P < 0.05, **P < 0.01, ***P <0.001. **Fig S4.** IFN-α depresses the succinylation of histone H3K79 on HBV cccDNA minichromosome. (a) HepaRG cells were infected with HBV at an MOI of 600 vp/cell and treated with IFN-α for 3 days at the indicated dose. The levels of histone H3K27 acetylation and histone H3K79 succinylation were measured by Western blot analysis in the cells. (b) Histone H3K27 acetylation and histone H3K79 succinylation on cccDNA were assessed by ChIP-qPCR in the cells. (c) HepaRG cells with depleted endogenous GCN5 and reconstituted expression of wild-type flag-GCN5 or flag-GCN5Y645mut were infected with HBV at an MOI of 600 vp/cell and treated with IFN-α for 3 days at the indicated dose. Histone H3K79 succinylation on cccDNA was assessed by ChIP-qPCR in the cells. (d, e, f) The levels of HBeAg, HBsAg, and HBV DNA were analysed by ELISA and qPCR assays in the supernatant of medium from HepaRG cells. The mean ± SD of at least three experiments is shown. Statistically significant differences are indicated as follows: **P<0.01; ***P<0.001. **Fig S5.** The original raw images of Western blot analysis (a-f) and Southern blot analysis (g) in figures or supplementary figures.

## Data Availability

Not applicable.

## Abbreviations

HBV: Hepatitis B virus
cccDNA: Covalently closed circular DNA
Ksucc: Lysine succinylation
pgRNA: Pregenomic RNA
rcDNA: Relaxed circular DNA
HBc: Virally encoded core antigen
HBx: Hepatitis B virus X protein
IFN-α: Interferon-α
PRC2: Polycomb repressive complex 2
PTMs: Posttranslational modifications
Kac: Lysine acetylation
KAT2A: Lysine acetyltransferase 2A
GCN5: Lysine acetyltransferase 2A
GNAT: A member of the GCN5-related N-acetyltransferase
HAT: Histone acetyltransferase
