# Supplementary material for: IFN-α confers epigenetic regulation of HBV cccDNA minichromosome by modulating GCN5-mediated succinylation of histone H3K79 to clear HBV cccDNA
Source: Clin Epigenetics. 2020 Sep 7;12:135. doi: 10.1186/s13148-020-00928-z (PMC7487718; doi:10.1186/s13148-020-00928-z)
Supplement: Supplementary file 1 — Additional file 1: Supplementary information. Table S1. List of antibodies used in this paper. Table S2. The characteristics of paracancerous liver tissues of HCC patients. Table S3. List of primers used in this paper. Fig S1. Succinylation of histone H3K79 contributes to the transcription of HBV DNA. (a) The efficiency of H3 overexpression was validated by Western blot analysis in HepaRG and HepG2 cells. (b) The efficiency of knockdown and overexpression of GCN5 was validated by Western blot analysis in HepaRG and HepG2 cells. (c-e) shGCN5 was transduced into HBV-infected HepaRG cells in 100-mm dishes for 5 days. GCN5 or GCN5Y645mut was transduced into HBV-infected HepaRG cells in 100-mm dishes for 6 days. The cells were harvested and used for ChIP assays with the indicated antibodies. Levels of the specific proteins on HBV cccDNA were analysed by ChIP-qPCR. The mean ± SD of at least three experiments is shown. *P < 0.05, **P < 0.01. Abbreviation: N.S., not significant. Fig S2. GCN5 is responsible for the succinylation of histone H3K79 on HBV cccDNA minichromosome. The efficiency of siGCN5 was validated by Western blot analysis in 293T, HepaRG and HepG2 cells. Fig S3. Relative mRNA levels of GCN5 were detected by RT-qPCR in cccDNA-positive paracancerous liver tissues (n=15) and cccDNA-negative paracancerous liver tissues (n=15). *P < 0.05, **P < 0.01, ***P <0.001. Fig S4. IFN-α depresses the succinylation of histone H3K79 on HBV cccDNA minichromosome. (a) HepaRG cells were infected with HBV at an MOI of 600 vp/cell and treated with IFN-α for 3 days at the indicated dose. The levels of histone H3K27 acetylation and histone H3K79 succinylation were measured by Western blot analysis in the cells. (b) Histone H3K27 acetylation and histone H3K79 succinylation on cccDNA were assessed by ChIP-qPCR in the cells. (c) HepaRG cells with depleted endogenous GCN5 and reconstituted expression of wild-type flag-GCN5 or flag-GCN5Y645mut were infected with HBV at an MOI of 600 vp/c [file 13148_2020_928_MOESM1_ESM.zip › Table S2.doc]

**Table S2: The characteristics of non-tumor liver tissues of HCC patients**

| **No.** | **Age** | **Gender** | **Organ** | **Chronic hepatitis with HBV** | **HBV DNA** | **cccDNA** |
| --- | --- | --- | --- | --- | --- | --- |
| 1 | 59 | F | Liver | + | + | + |
| 2 | 60 | M | Liver | + | + | - |
| 3 | 64 | M | Liver | + | + | + |
| 4 | 67 | M | Liver | + | + | + |
| 5 | 43 | F | Liver | + | + | - |
| 6 | 41 | M | Liver | + | + | + |
| 7 | 45 | M | Liver | + | + | + |
| 8 | 56 | M | Liver | + | + | - |
| 9 | 70 | M | Liver | + | + | - |
| 10 | 67 | M | Liver | + | + | + |
| 11 | 59 | M | Liver | + | + | + |
| 12 | 57 | M | Liver | + | + | + |
| 13 | 61 | F | Liver | + | + | - |
| 14 | 51 | F | Liver | + | + | + |
| 15 | 56 | M | Liver | + | + | + |
| 16 | 60 | M | Liver | + | + | - |
| 17 | 54 | M | Liver | + | + | + |
| 18 | 36 | M | Liver | + | + | - |
| 19 | 46 | M | Liver | + | + | + |
| 20 | 60 | M | Liver | + | + | + |
| 21 | 59 | M | Liver | + | + | - |
| 22 | 57 | F | Liver | + | + | + |
| 23 | 51 | M | Liver | + | + | - |
| 24 | 38 | M | Liver | + | + | + |
| 25 | 56 | M | Liver | + | + | - |
| 26 | 49 | M | Liver | + | + | + |
| 27 | 58 | M | Liver | + | + | - |
| 28 | 46 | F | Liver | + | + | + |
| 29 | 56 | M | Liver | + | + | + |
| 30 | 54 | M | Liver | + | + | - |
| 31 | 39 | M | Liver | + | + | - |
| 32 | 42 | M | Liver | + | + | + |
| 33 | 53 | F | Liver | + | + | + |
| 34 | 60 | M | Liver | + | + | - |
| 35 | 66 | M | Liver | + | + | + |
| 36 | 60 | M | Liver | + | + | - |
| 37 | 59 | M | Liver | + | + | + |
| 38 | 69 | M | Liver | + | + | + |
| 39 | 38 | F | Liver | + | + | + |
| 40 | 56 | M | Liver | - | - | - |
| 41 | 53 | M | Liver | + | - | - |
| 42 | 47 | M | Liver | - | - | - |
| 43 | 67 | M | Liver | - | - | - |
